# Lack of BRAFV600E mutation in stage I and II of colorectal cancer 

**Published:** 2016

**Authors:** Mahsa Molaei, Roya Kishani Farahani, Mina Maftouh, Mohammad Yaghoob Taleghani, Mahsa Vahdatinia, Fatemeh Khatami, Ehsan Nazemalhosseini- Mojarad, Hamid Asadzadeh Aghdae, Akram Aboutorabi, Mohammad Reza Zali

**Affiliations:** 1 *Gastroenterology and Liver Disease Research center, Research Institute for Gastroenterology and liver Disease, Shahid Beheshti University of Medical Sciences, Tehran, Iran.*; 2 *Basic and molecular epidemiology of Gastrointestinal disorders Research center, Research Institute for Gastroenterology and liver disease, Shahid Beheshti University of Medical Sciences, Tehran, Iran *; 3 *Student Research Committee, School of Pharmacy, Shahid Beheshti University of Medical Sciences, Tehran, Iran *

**Keywords:** BRAFV600E mutation, CRC, DNA sequencing

## Abstract

**Aim:**

We aimed to explore the frequency of BRAFV600E mutation in Iranian patients with colorectal cancer (CRC) as well as its association with clinic pathological characteristic of patients.

**Background:**

CRC is the third leading cause of cancer related death. There is a growing body of data showing the association of BRAFV600E mutation with malignant transformation and clinical outcome of different tumors, including CRC. These findings suggest that BRAFV600E mutation can be used as diagnostic and/or prognostic biomarker for management of cancer patients.

**Patients and methods:**

A total of 85 patients with sporadic tumor were recruited. BRAFV600E mutation was investigated using sequencing of extracted DNAs from formalin-fixed paraffin-embedded (FFPE) tumor tissues. Electropherograms were analyzed using Laser-gene 6 software.

**Results:**

More than 95% of patients were in stage I and II and none of them were in stage IV. Patients were mostly below 55 years old and tumors were dominantly located in the distal colon. Of note, no BRAFV600E mutations were detected in our population.

**Conclusion:**

Our results showed no V600E mutation in the BRAF gene in stage I and II of CRC patients. Further studies in multi-center settings are warranted to examine the prognostic and/or predictive value of this marker in different stages of colorectal cancer patients.

## Introduction

 Colorectal cancer (CRC) is the third-most-common form of cancer in the world (1). Several genetic mutations have been reported to be associated with the development and progression of this disease, including V-Raf Murine Sarcoma Viral Oncogene Homolog B1 (BRAF). BRAF is one of the main genes, involved in the progression of several malignancies, including CRC (2, 3). This gene encodes cytoplasmic serine-thereonine kinase that mediates intracellular signaling through the RAS/RAF/MAPK pathway (4). The mitogen activated protein kinase (MAPK) pathway is one of the most critical pathways in the regulation of cancer cell proliferation and survival (5). Oncogenic mutations in BRAF cause constitutive activation of MAPK pathway (6). More than 80% of mutation are found in exon 15 at nucleotide position 1799, causing thymine to adenine transversion within codon 600 leading to substitution of valine by glutamic acid at protein level (2, 7). Activating BRAF mutations have been identified in 4% to 12% of unselected colorectal tumors in all Dukes’ stages (8-13). Moreover, this mutation has been reported in several forms of premalignant lesions, such as adenomas, hyperplastic polyps, and serrated adenomas (5-6), as well as in aberrant crypt foci (14, 15). BRAF gene is usually present as wild type in hereditary non-polyposis colorectal cancer (HNPCC), whereas the BRAFV600E mutation is often found in the sporadic CRC (16-18). Several studies have shown that BRAFV600E mutation is associated with survival in metastatic CRC patients, suggesting its prognostic value (19). BRAFV600E mutation is also being suggested as a predictive biomarker in response to anti-EGFR drug therapy. In particular, patients harboring this mutation were resistant to Cetuximab and panitumumab drugs (19). Due to the crucial role of BRAFV600E mutation in diagnostic, therapeutic and prognostic fields, in the present study, we further investigate the prognostic value of this marker with clinical outcome of 85 CRC patients. 

## Patients and Methods

***Patients***

This retrospective cross sectional study was performed on 85 randomly selected patients with colorectal cancer, who had undergone surgical resection of adenocarcinoma and referred to the gastroenterology and liver disease research center of Shahid Beheshti University of Medical Sciences (Tehran, Iran) from June 2013 to December 2014. Patients with Familial Adenomatous Polyposis coli (FAP) or hereditary non-polyposis CRC (HNPCC) were excluded. Informed consent was obtained from all the patients, or their relatives. Demographic and clinical information of the patients were obtained; including: age, sex, tumor location, tumor grade, smoking status and alcohol consumption. Tumor tissue specimens were harvested from tumor mass after surgical resection. 

***Deoxyribonucleic acid (DNA) extraction***

DNA was extracted from paraffin-embedded tissues using the Qiagen kit. The quality of the extracted DNA was assessed by NanoDrop spectrophotometer (NanoDrop Technologies, Inc., Wilmington, DE, USA). 

***DNA sequencing***

The 160 bp DNA fragment of the exon 15 of BRAF gene was amplified using specific primers (forward 5'GCTTGCTCTGATAGGAAAATGAGATC3' and reverse 5'ATCCAGACAACTGTTCAAACTGATG3'), as described previously (20). To observe the digested fragments, the PCR products were separated in 2% agarose gel and stained with ethidium bromide. Direct sequencing was performed, using fluorescent dideoxy on ABI sequencing 3130 XL, according to manufacturer instructions. Samples were then subjected to direct sequencing of single strand PCR product using Big Dye Terminator v 3cycle sequencing kit and the ABI 3130XL genetic analyzer (Applied Biosystems). The electropherograms were processed using Lasergene software version 6 (DNA star).

***Statistical analysis***

Statistical analysis was performed using the SPSS software program for Windows, Release 13.0.0 (SPSS Inc., Chicago, IL). Comparison of variables was performed using Pearson’s Chi-square test, Fisher’s exact test, or the Mann-Whitney U test, depending on the nature of the data. Two-tailed p <0.05 was considered statistically significant. 

## Results

***Demographic and Clinical characteristic of population***

A total of 85 colorectal cancer patients were enrolled in the present study. Demographic and clinicopathological features of patients are summarized in Table 1. Most of patients were male, non-smokers and non-alcoholic. Age distribution was more frequent in younger patients (52.9%) and tumors locations were dominant in distal colon (54.1%), compared to proximal colon (45.8%)**. **More than 95% of patients were in stage I and II, while there were no patients in stage IV.

***BRAFV600E mutation status of the patients***

To evaluate the mutation status of our patients for BRAFV600E, direct sequencing was carried out in genomic DNA extracted from FFPE of all cases, using the fluorescent dideoxy method. Our analyses illustrated that none of patients had BRAFV600E mutation. 

## Discussion

Colorectal cancer is a heterogeneous disease with multiple underlying genetic mutations, leading to the development of this disease (21-25). Mutations in BRAF gene have been documented to be involved in malignant transformation of tumor tissue. Several studies have reported the frequency of BRAFV600E mutation in CRC between 0 and 23% in different populations. In particular, Fariña-Sarasqueta and colleagues investigated the value of BRAF, microsatellite instability (MSI) and KRAS mutations on clinical outcome of 106 stage II colon carcinoma patients undergone surgery and 258 stage III patients treated with 5-fluorouracil chemotherapy. They showed that the V600E BRAF mutation were associated with poor prognosis in CRC patients (26). Another study by Shaukate et al., examined the correlation of BRAFV600E mutation in 63 intervals versus 131 non-interval colorectal cancer patients. They found no association between this mutation and interval cancers, although they suggested its value as a marker of poor prognosis, particularly in microsatellite stable cancers (27). There is a growing body of evidence reporting the rate of BRAF mutation in CRC patients, such as in Switzerland (19.7%) (28), Netherland (18.7%) (26), USA (21.8%) (27), Italy (14.9%) (29), France (4.3%) (30), and Belgium (4.7%) (31). A low incidence of BRAF mutation in CRC was observed in Asians e.g. Thailand (0%) (32), Saudi Arabia (2.5%) (33), China (4.9%) (34), Taiwan (1%) (35), and Japan (4.7%) (36). This can be explained at least in part by ethnicity. Thus, we conducted a cross sectional retrospective study to determine the rate of BRAFV600E mutation in an Iranian population with colorectal cancer. We also investigated the association of this mutation with clinicopathological features of cases, including tumor location, grade, and demographic data such as age, gender, smoking and alcohol consumption. Our data showed enrolled patients in this study didn’t have any mutation in BRAF gene. In line of our observation Naghibalhossaini et al. showed the absence of BRAF mutation among the Iranian population (24). Additionally Brim et al., reported a very low rate of mutation in BRAF among CRC patients in Iran (2 %) (22). 

**Table 1:** Characteristic of colorectal cancer patients

Characteristic		Number of patients	Percentage
Sex	Male	48	56.4
	Female	37	43.5
Age at diagnosis	< 50 year	45	52.9
	≥ 50 year	40	47
Tumor Site	Proximal	39	45.8
	Distal	46	54.1
Tumor Grade	I	48	56.4
	II	33	38.8
	III	4	4.7
	IV	0	0
Smoking	Current user	11	12.9
	Never user	62	72.9
	Previous user	12	14.1
Alcohol	Current user	4	4.7
	Never user	75	88.2
	Previous user	5	5.8

On the other hand, a recent study revealed that BRAF mutation is more common in older (>60yr) and female patients (37). However, most of our cases were below 55 years old, which might explain the lack of this mutation in our cases. Moreover, some studies reported a relationship between the history of smoking in CRC patients with BRAFV600E mutation (38-40). Their results showed a negative correlation between BRAFV 600E mutation and alcohol intake. Our patients participated in this study were non-smokers and non-alcoholic. This might be another reason for the lack of BRAF mutation in our cases. In addition, this mutation was found mostly in patients with stage III, although our subjects were in stage I and II. This could explain that BRAF mutation might be present in late stages. 

In conclusion, our data showed the lack of BRAF mutation in stage I and II of CRC patients, supporting further studies in a larger population and multi center setting to evaluate the prognostic and/or predictive value of BRAF mutation in CRC patients.

## Note

**(**Please cite as:** Molaei** **M, Kishani Farahani** **R, Maftouh** **M, Taleghani** **MY, Vahdatinia** **M, Khatami F, et al. Lack of BRAFV600E mutation in stage I and II of colorectal cancer. Gastroenterol Hepatol ****Bed Bench**** 2016;9(2):94-99).**

## References

[1] Calvert PM, Frucht H (2002). The genetics of colorectal cancer. Ann Intern Med.

[2] Davies H, Bignell GR, Cox C, Stephens P, Edkins S, Clegg S (2002). Mutations of the BRAF gene in human cancer. Nature.

[3] Brose MS, Volpe P, Feldman M, Kumar M, Rishi I, Gerrero R (2002). BRAF and RAS mutations in human lung cancer and melanoma. Cancer Res.

[4] Marais R, Marshall CJ (1996). Control of the ERK MAP kinase cascade by Ras and Raf. Cancer Surv.

[5] Kyriakis JM, App H, Zhang XF, Banerjee P, Brautigan DL, Rapp UR (1992). Raf-1 activates MAP kinase-kinase. Nature.

[6] Malumbres M, Barbacid M (2003). RAS oncogenes: the first 30 years. Nat Rev Cancer.

[7] Wan PT, Garnett MJ, Roe SM, Lee S, Niculescu-Duvaz D, Good VM (2004). Mechanism of activation of the RAF-ERK signaling pathway by oncogenic mutations of B-RAF. Cell.

[8] Rajagopalan H, Bardelli A, Lengauer C, Kinzler KW, Vogelstein B, Velculescu VE (2002). Tumorigenesis: RAF/RAS oncogenes and mismatch-repair status. Nature.

[9] Nagasaka T, Sasamoto H, Notohara K, Cullings HM, Takeda M, Kimura K (2004). Colorectal cancer with mutation in BRAF, KRAS, and wild-type with respect to both oncogenes showing different patterns of DNA methylation. J Clin Oncol.

[10] Kambara T, Simms LA, Whitehall VL, Spring KJ, Wynter CV, Walsh MD (2004). BRAF mutation is associated with DNA methylation in serrated polyps and cancers of the colorectum. Gut.

[11] Yuen ST, Davies H, Chan TL, Ho JW, Bignell GR, Cox C (2002). Similarity of the phenotypic patterns associated with BRAF and KRAS mutations in colorectal neoplasia. Cancer Res.

[12] Deng G, Bell I, Crawley S, Gum J, Terdiman JP, Allen BA (2004). BRAF mutation is frequently present in sporadic colorectal cancer with methylated hMLH1, but not in hereditary nonpolyposis colorectal cancer. Clin Cancer Res.

[13] Miyaki M, Iijima T, Yamaguchi T, Kadofuku T, Funata N, Mori T (2004). Both BRAF and KRAS mutations are rare in colorectal carcinomas from patients with hereditary nonpolyposis colorectal cancer. Cancer Lett.

[14] Beach R, Chan AO, Wu TT, White JA, Morris JS, Lunagomez S (2005). BRAF mutations in aberrant crypt foci and hyperplastic polyposis. Am J Pathol.

[15] Vandrovcova J, Lagerstedt-Robinsson K, Pahlman L, Lindblom A (2006). Somatic BRAFV600E Mutations in Famlial Colorectal Cancer. Cancer Epidemiol Biomarkers Prev.

[16] Capper D, Voigt A, Bozukova G, Ahadova A, Kickingereder P, von Deimling A (2013). BRAFV600E specific immunohistochemistry for the exclusion of Lynch syndrome in MSI-H colorectal cancer. Int J Cancer.

[17] Toon CW, Walsh MD, Chou A, Capper D, Clarkson A, Sioson L (2013). BRAFV600E immunehistochemistry facilitates universal screening of colorectal cancers for Lynch syndrome. Am J Surg Pathol.

[18] Thiel A, Heinonen M, Kantonen J, Gylling A, Lahtinen L, Korhonen M (2013). BRAF mutation in sporadic colorectal cancer and Lynch syndrome. Virchows Arch.

[19] Thiel A, Ristimäki A (2013). Toward a molecular classification of colorectal cancer: the role of BRAF. Front Oncol.

[20] Benlloch S, Payá A, Alenda C, Bessa X, Andreu M, Jover R (2006). Detection of BRAFV600E Mutation in Colorectal Cancer. J Mol Diagn.

[21] Kalady MF, Dejulius KL, Sanchez JA, Jarrar A, Liu X, Manilich E (2012). BRAF mutation in colorectal cancer are associated with distinct clinical characteristics and worse prognosis. Dis Colon Rectum.

[22] Brim H, Mokarram P, Naghibalhossaini F, Saberi-Firoozi M, Al-Mandhari M, Al-Mawaly K (2008). Impact of BRAF, MLH1 on the incidence of microsatellite instability high colorectal cancer in populations based study. Mol Cancer.

[23] Nazemalhosseini Mojarad E, Farahani RK, Haghighi MM, Aghdaei HA, Kuppen PJ, Zali MR (2013). et al. Clinical implications of BRAF mutation test in colorectal cancer. Gastroenterol Hepatol Bed Bench.

[24] Naghibalhossaini F, Mahmoodzadeh Hosseini H, Mokarram P, Zamani M (2011). High frequency of genes’ promoter methylation, but lack of BRAFV600E mutation among Iranian colorectal cancer patients. Pathol Oncol Res.

[25] Nazemalhosseini Mojarad E, Kuppen PJ, Aghdaei HA, Zali MR (2013). The CpG island methylator phenotype (CIMP) in colorectal cancer. Gastroenterol Hepatol Bed Bench.

[26] Fariña-Sarasqueta A, van Lijnschoten G, Moerland E, Creemers GJ, Lemmens VE, Rutten HJ (2010). The BRAFV600E mutation is an independent prognostic factor for survival in stage II and stage III colon cancer patients. Ann Oncol.

[27] Shaukat A, Arain M, Thaygarajan B, Bond JH, Sawhney M (2010). Is BRAF mutation associated with interval colorectal cancers?. Dig Dis Sci.

[28] Zlobec I, Bihl MP, Schwarb H, Terracciano L, Lugli A (2010). Clinicopathological and protein characterization of BRAF- and K-RAS-mutated colorectal cancer and implications for prognosis. Int J Cancer.

[29] Loupakis F, Ruzzo A, Cremolini C, Vincenzi B, Salvatore L, Santini D (2009). KRAS codon 61, 146 and BRAF mutations predict resistance to cetuximab plus irinotecan in KRAS codon 12 and 13 wild-type metastatic colorectal cancer. Br J Cance.

[30] Laurent-Puig P, Cayre A, Manceau G, Buc E, Bachet JB, Lecomte T (2009). Analysis of PTEN, BRAF, and EGFR status in determining benefit from cetuximab therapy in wild-type KRAS metastatic colon cancer. J Clin Oncol.

[31] De Roock W, Claes B, Bernasconi D, De Schutter J, Biesmans B, Fountzilas G (2010). Effects of KRAS, BRAF, NRAS, and PIK3CA mutations on the efficacy of cetuximab plus chemotherapy in chemotherapy-refractory metastatic colorectal cancer: a retrospective consortium analysis. Lancet Oncol.

[32] Chaiyapan W, Duangpakdee P, Boonpipattanapong T, Kanngern S, Sangkhathat S (2013). Somatic mutations of K-ras and BRAF in Thai colorectal cancer and their prognostic value. Asian Pac J Cancer Prev.

[33] Siraj AK, Bu R, Prabhakaran S, Bavi P, Beg S, Al Hazmi M (2014). A very low incidence of BRAF mutations in Middle Eastern colorectal carcinoma. Mol Cancer.

[34] Chen J, Guo F, Shi X, Zhang L, Zhang A, Jin H (2014). BRAFV600E mutation and KRAS codon 13 mutations predict poor survival in Chinese colorectal cancer patients. BMC Cancer.

[35] Hsieh LL, Er TK, Chen CC, Hsieh JS, Chang JG, Liu TC (2012). Characteristics and prevalence of KRAS, BRAF, and PIK3CA mutations in colorectal cancer by high-resolution melting analysis in Taiwanese population. Clin Chim Acta.

[36] Asaka S, Arai Y, Nishimura Y, Yamaguchi K, Ishikubo T, Yatsuoka T (2009). Microsatellite instability-low colorectal cancer acquires a KRAS mutation during the progression from Dukes' A to Dukes' B. Carcinogenesis.

[37] Kin C, Kidess E, Poultsides GA, Visser BC, Jeffrey SS (2013). Colorectal cancer diagnostics: biomarkers, cell-free DNA, circulating tumor cells and defining heterogeneous populations by single-cell analysis. Expert Rev Mol Diagn.

[38] Curtin K, Samowitz WS, Wolff RK, Herrick J, Caan BJ, Slattery ML (2009). Somatic alterations, metabolizing genes and smoking in rectal cancer. Int J Cancer.

[39] Samowitz WS, Albertsen H, Sweeney C, Herrick J, Caan BJ, Anderson KE (2006). Association of smoking, CpG island methylator phenotype, and V600E BRAF mutations in colon cancer. J Natl Cancer Inst.

[40] Chen D, Huang JF, Liu K, Zhang LQ, Yang Z, Chuai ZR (2014). BRAFV600E Mutation and Its Association with Clinicopathological Features of Colorectal Cancer: A Systematic Review and Meta-Analysis. PLoS One.

